# AtDsPTP1 acts as a negative regulator in osmotic stress signalling during *Arabidopsis* seed germination and seedling establishment

**DOI:** 10.1093/jxb/eru484

**Published:** 2014-12-24

**Authors:** Rui Liu, Yinggao Liu, Nenghui Ye, Guohui Zhu, Moxian Chen, Liguo Jia, Yiji Xia, Lu Shi, Wensuo Jia, Jianhua Zhang

**Affiliations:** ^1^College of Life Science, Shandong University, Jinan, Shandong, China; ^2^Department of Biology, Hong Kong Baptist University, Hong Kong, China; ^3^State Key Laboratory of Crop Biology, College of Life Science, Shandong Agricultural University, Taian, Shandong, China; ^4^Shenzhen Research Institute, The Chinese University of Hong Kong, Shenzhen, China; ^5^College of Life Sciences, South China Agricultural University, Guangdong, China; ^6^School of Life Science and State Key Laboratory of Agrobiotechnology, The Chinese University of Hong Kong, Hong Kong, China; ^7^College of Agronomy and Biotechnology, China Agricultural University, Beijing, China

**Keywords:** Abscisic acid (ABA), *Arabidopsis*, dual-specificity protein phosphatase, osmotic stress, root elongation, seed germination.

## Abstract

Dual-specificity protein phosphatases (DsPTPs) target both tyrosine and serine/threonine residues and play roles in plant growth and development. We have characterized an *Arabidopsis* mutant, *dsptp1*, which shows a higher seed germination rate and better root elongation under osmotic stress than the wild type. By contrast, its overexpression line, *DsPTP1-OE*, shows inhibited seed germination and root elongation; and its complemented line, *DsPTP1-Com*, resembles the wild type and rescues *DsPTP1-OE* under osmotic stress. Expression of *AtDsPTP1* is enhanced by osmotic stress in seed coats, bases of rosette leaves, and roots. Compared with the wild type, the *dsptp1* mutant shows increased proline accumulation, reduced malondialdehyde (MDA) content and ion leakage, and enhanced antioxidant enzyme activity in response to osmotic stress. AtDsPTP1 regulates the transcript levels of various dehydration-responsive genes under osmotic stress. Abscisic acid (ABA) accumulation in *dsptp1* under osmotic stress is reduced with reduced expression of the ABA-biosynthesis gene *NCED3* and increased expression of the ABA-catabolism gene *CYP707A4*. *AtDsPTP1* also regulates the expression of key components in the ABA-signalling pathway. In conclusion, AtDsPTP1 regulates ABA accumulation, and acts as a negative regulator in osmotic stress signalling during *Arabidospsis* seed germination and seedling establishment.

## Highlight

AtDsPTP1 was found to regulate ABA accumulation and act as a negative regulator in osmotic stress signalling during *Arabidospsis* seed germination and seedling establishment.

## Introduction

The signalling pathways of plants under osmotic stress have been intensively studied during the past two decades (Hasegawa *et al.*, 2000; Xiong *et al.*, 2002; Xiong and Zhu, 2002; Fujii and Zhu, 2012). Different protein kinases and transcription factors reportedly minimize damage caused by osmotic stress (Droillard *et al.*, 2004; Gilmour *et al.*, 2004). Abscisic acid (ABA) synthesis is significantly induced by the stress and its signalling has a crucial function in abiotic stresses responses (Agarwal and Jha, 2010; Fujita *et al.*, 2011). The core module of the ABA-signalling pathway in response to osmotic stress, as well as other abiotic stresses, has been elucidated (Ye *et al.*, 2012). However, many components in the signalling network of osmotic stress remain unknown (Cutler *et al.*, 2010). Further analysis is needed to determine the exact functions and connections among the components involved in the regulation of plant responses to osmotic stress.

Similar to protein kinases, protein phosphatases (PPs) are also indispensable in stress signalling. According to their dephosphorylation targets, PPs can be divided into two groups, namely serine/threonine phosphatases and protein tyrosine phosphatases (PTPs), both of which have key functions in plant growth regulation and abiotic stresses responses (Schweighofer *et al.*, 2004; Schweighofer and Meskiene, 2008; Bartels *et al.*, 2010). Dual-specificity protein phosphatases (DsPTPs) can target both tyrosine and serine/threonine (Keyse, 1995). Except for the active site motif, DsPTPs do not share sequence similarity with PTPs. DsPTPs often have strict substrate specificity, and each member usually acts on only one or a limited number of target proteins (Keyse, 1995). In *Arabidopsis*, different DsPTPs and their associated protein kinases control plant growth and development under stress conditions. For example, mutation of the *MKP1* gene results in plant hypersensitivity to stresses imposed by ultraviolet light and methyl methanesulfonate (Ulm *et al.*, 2001). By contrast, *mkp1* plants show hyposensitivity to salt stress (Ulm *et al.*, 2002). PHS1, a DsPTP, controls microtubule organization and embryonic development (Naoi and Hashimoto, 2004). Furthermore, *AtPTEN1*, which encodes another DsPTP, is expressed exclusively in pollen and is essential for pollen development (Gupta *et al.*, 2002). These studies suggest that DsPTPs have diverse functions in different biological processes.

Increasing evidence has shown that many protein kinases have positive functions in the osmotic stress-signalling pathway (Fujii and Zhu, 2012). However, PPs are also involved in the signalling process. In alfalfa, MP2C, a PP2C, suppresses the lethal phenotype induced by STE11, which is involved in the hyperosmotic HOG1 cascade (Meskiene *et al.*, 1998). Furthermore, MP2C dephosphorylates and inactivates the stress-activated SIMK (Meskiene *et al.*, 2003). In *Arabidopsis*, AtPTP1 dephosphorylates AtMAPK4 and AtMAPK6 *in vitro*, leading to the inactivation of these kinases that participate in osmotic stress signalling (Huang *et al.*, 2000; Gupta and Luan, 2003). Two *Arabidopsis* PP2Cs, ABI1 and ABI2, negatively regulate ABA signalling in drought-induced stomatal closure (Schweighofer *et al.*, 2004). However, the function of DsPTPs in osmoregulation is still unknown. One report on the identification of the *mkp1* mutant, which exhibits enhanced salt stress tolerance, indicates that the DsPTPs have critical functions in osmoregulation (Droillard *et al.*, 2004). Investigation of the functions of AtDsPTPs in response to osmotic stress is of great significance.

The *AtDsPTP1* gene of *Arabidopsis* was identified more than a decade ago. The AtDsPTP1 protein is an active phosphatase which can inactivate AtMPK4 protein *in vitro* (Gupta *et al.*, 1998). However, little is known about its biological functions under osmotic stress. In this study, the *dsptp1* mutant shows enhanced tolerance to osmotic stress during seed germination and seedling establishment. This DsPTP positively regulates ABA accumulation and its signalling pathway in response to osmotic stress.

## Materials and methods

### Plant materials and growth conditions

The T-DNA insertion mutant *dsptp1* (SALK_092811) was obtained from the Arabidopsis Biological Resource Centre (http://www.arabidopsis.org/). It was grown and screened for homozygosity. All seeds were of the Columbia ecotype: mutant, WT, overexpression (OE), and complemented (Com). *Arabidopsis thaliana* L. plants were kept in a growth chamber with a 16h photoperiod at a photon flux density of ~200 µmol m^–2^ s^–1^ at day/night temperature of 23/20°C, and a relative humidity of 90%.

### Stress and ABA treatments

Two-week-old *Arabidopsis* seedlings on MS plates were transferred to filter papers saturated with different concentrations of mannitol or ABA solution and incubated for different times.

### Seed germination assays

WT, *dsptp1*-mutant, OE, and Com line seeds (>100 seeds for each replicate) were surface sterilized. The seeds were sown on MS medium (Murashige and Skoog, 1962; 1 × MS salts, 10g l^–1^ sucrose, and 10g l^–1^ agar, pH 5.7) with or without either different osmotic stress-generator solutions or ABA solutions and then incubated at 23°C with a 16h light photoperiod. The number of planted and germinated seeds was recorded 7 d after being sown on the medium. Radicle emergence of >1mm indicated seed germination. Three replicate plates were used for each treatment.

### Root elongation measurements

*Arabidopsis* seeds were sown on MS medium as above, stratified for 3d, and then incubated at 23°C for 7 d. For root elongation measurements, 15 seeds were used per replicate, and three replicates were made for each treatment. Seven-d-old seedlings with roots 1–1.5cm long were transferred from agar plates containing MS medium onto a second agar medium supplemented with different concentrations of osmotic stress-generator. Increases in root length were measured after 7 d of treatment (Rosado *et al.*, 2006).

### Histochemical analysis of GUS activity

A DNA fragment containing 1139bp of the upstream region of the *ATDsPTP1* gene was amplified by PCR using oligonucleotides incorporating XbaI or BamHI sites at the 5’ ends [5’-TG*TCTAGA*CCTTGGTTTCTGTTACTTGGCTC-3’

(XbaI site underlined) and 5′- AA*GGATCC*GAAGGA ATACGAATT CGCCAAAG-3′ (BamHI site underlined)]. The PCR fragments were digested with XbaI and BamHI and then cloned into the XbaI-BamHI site of a promoterless GUS expression vector, pBI101.3 (Clontech). The resulting construct, *ATDsPTP1pro-GUS*, was transferred from *Escherichia coli* DH5α into *Agrobacterium tumefaciens* GV3101. *Arabidopsis* was transformed by vacuum infiltration with *Agrobacterium* containing *ATDsPTP1pro-GUS*. Following the methods described by Bechtold *et al.* (1993), histochemical localization of independent transgenic lines was performed in 5-bromo-4-chloro-3-indolyl glucuronide (X-gluc) buffer (50mM sodium phosphate buffer, pH 7.0, 10mM EDTA, 0.1% Triton X-100, 2% DMSO, 0.5mM potassium ferrocyanide, 2mg ml^–1^ X-gluc) at 37°C for 6–12h. The stained plants were washed for 30min with 50–100% ethanol to remove the chlorophyll.

### Isolation of total RNA and quantitative real-time RT-PCR

Two-week-old plants were transferred from MS agar plates to Petri dishes and placed on a filter paper soaked with 330mM mannitol solution osmotic treatment. Total RNA was isolated from *Arabidopsis* seeds or leaves using an RNeasy Plant mini kit (Qiagen, Valencia, CA, USA) according to the manufacturer’s instructions. A 2 μg aliquot of RNA was reverse transcribed to cDNA with a SuperScript III RTS first-strand cDNA synthesis kit (Invitrogen, Carlsbad, CA, USA). Transcript levels of each gene were measured by quantitative real-time RT-PCR (qRT-PCR) using an iCycler (Bio-Rad, Hercules, CA, USA) with iQ SYBR Green Supermix (Bio-Rad). To standardize the results, the amplification of *AtACTIN* was also determined and used as the internal standard. The data were normalized to the amplification of an *Arabidopsis* actin gene. For each sample, the mean value from three qRT-PCRs was used to calculate transcript abundance. The mean values were then plotted with their standard errors. The primers that were used in qRT-PCR are summarized in Supplementary Table S1.

### Antioxidant enzyme assays

Frozen leaf segments (0.3g) were homogenized on ice with 1ml of 50mM potassium phosphate buffer (pH 7.0), containing 1mM EDTA and 1% polyvinylpyrrolidone (PVP). The buffer was mixed with 1mM ascorbate for the ascorbate peroxidase (APX) assay. The homogenate was centrifuged at 15 000*g* for 20min at 4°C and the supernatant was immediately used for the following antioxidant enzyme assays. Protein content was determined according to the method of Bradford (1976) with bovine serum albumin (BSA) as the standard.

The total activities of antioxidant enzymes were determined as described previously (Jiang and Zhang, 2001). Total superoxide dismutase (SOD) (EC 1.15.1.1) activity was assayed by monitoring the inhibition of photochemical reduction of nitroblue tetrazolium (NBT). The 2.5ml reaction mixture contained 50mM potassium phosphate buffer (pH 7.8), 13mM methionine, 75mM NBT, 2mM riboflavin, 0.1mM EDTA, and 20 µl of enzyme extract. The reaction mixtures were illuminated for 15min at a light intensity of 5000 lux. One unit of SOD activity was defined as the amount of enzyme required to cause 50% inhibition of the reduction of NBT as monitored at 560nm.

Total catalase (CAT) (EC 1.11.1.6) activity was assayed by measuring the decomposition rate of H_2_O_2_ (extinction coefficient 39.4mM^–1^ cm^–1^) at 240nm for 1min. The reaction mixture contained 50mM potassium phosphate buffer (pH 7.0), 10mM H_2_O_2_, and 20 µl of enzyme extract in a 3ml volume (Jiang and Zhang, 2001).

Total APX (EC 1.11.1.11) activity was measured by monitoring the decrease in absorbance at 290nm (extinction coefficient 2.8mM^–1^ cm^–1^) for 30 s in 1ml of a reaction mixture containing 50mM potassium phosphate buffer (pH 7.0), 0.5mM ascorbate, 0.1mM H_2_O_2_, and 20 µl of enzyme extract. The reaction was started with the enzyme extract. Correction was done for the low, non-enzymatic oxidation of ascorbate by H_2_O_2_ (Jiang and Zhang, 2001).

Total glutathione reductase (GR) (EC 1.6.4.2) activity was measured by following the oxidation of NADPH at 340nm (extinction coefficient 6.2mM^–1^ cm^–1^) for 1min in 1ml of an assay mixture containing 50mM potassium phosphate buffer (pH 7.8), 2mM Na_2_EDTA, 0.15mM NADPH, 0.5mM oxidized glutathione, and 100 µl of enzyme extract. The reaction was initiated by adding NADPH. Corrections were made for the background absorbance at 340nm without NADPH (Jiang and Zhang, 2001).

### Proline assays

Four-week-old *dsptp1* and WT (Col0) seedlings grown on soil were soaked with 330mM mannitol or 400mM sorbitol solution for different times. After treatment, the samples were frozen in liquid nitrogen and kept at –80°C for the proline assay. Proline concentration was determined following the methods described by Bates (1973).

### Malondialdehyde (MDA) measurements

Lipid peroxidation was evaluated according to the method of Heath and Packer (1968) with slight modification; the reaction solution was heated in boiling water for 15min. The MDA content was measured from 0.5g of two-week-old *Arabidopsis* seedlings. The results are expressed as μmol g FW^–1^ of seedlings and correspond to means of measurements carried out with four extracts.

### Ion leakage measurement

To measure ion leakage in mannitol-treated seedlings, two-week-old WT and *dsptp1* seedlings grown on MS agar plates were carefully removed from the plate, rinsed briefly with distilled water, and placed in solutions containing 550mM mannitol for different times. After treatment, seedlings were rinsed briefly with distilled water and placed immediately in a tube with 5ml of water. The tube was agitated gently for 3h before the electrolyte content was measured. Three repetitions of each treatment were conducted.

### Determination of endogenous ABA content

For the measurement of endogenous ABA, *Arabidopsis* leaves were ground in liquid nitrogen with the addition of 1ml of distilled water per 0.2g of frozen ground tissue, and then shaken at 4°C overnight. The homogenates were centrifuged at 12 000*g* for 20min at 4°C, and the supernatant was used directly for ABA assay. ABA analysis was carried out using RIA (radioimmune assay), as described by Quarrie *et al.* (1988). The reaction mixture (450 μl) contained 200 μl of phosphate buffer (pH 6.0), 100 μl of diluted antibody (Mac 252) (Abcam) solution (the antibody Mac252 was dissolved in PBS containing 5mg ml^–1^ BSA and 4mg ml^–1^ PVP), 100 μl of [^3^H]ABA (~8000 cpm) solution, and 50 μl of crude extract. The mixture was incubated at 4°C for 45min and bound radioactivity was measured in 50% saturated (NH_4_)_2_SO_4_-precipitated pellets with a liquid scintillation counter (Ye *et al.*, 2011).

### Generation of the DsPTP1-overexpressing (DsPTP1-OE) line and DsPTP1-complemented (DsPTP1-Com) line

Full-length *Arabidopsis DsPTP1* cDNA was obtained by using reverse transcription-PCR, cloned into the pENTR-TOPO cloning vector (Invitrogen) and sequenced. After the LR reaction, *DsPTP1* cDNA was inserted into the pGWB5 vector (a gift from Prof. Liang, YangZhou University) which had a 35S promoter; this vector was named pGWB5-*DsPTP1*. Transgenic *Arabidopsis* containing the cauliflower mosaic virus (CaMV) 35S promoter was generated using the floral-dipping method (Clough and Bent, 1998) and transferred into Col0 wild-type and *dsptp1*-mutant plants. Transformed plants were selected by growth on hygromycin-containing medium. Plants of the second generation after transformation were used for the experiments. The empty pGWB5 vector (the *ccdb* gene was substituted by a non-sense segment with a termination codon) which acted as control was also transferred into Col0 wild-type plants.

### Accession numbers

Sequence data from this article can be found in the *Arabidopsis* Genome Initiative or GenBank/EMBL databases under the following accession numbers: *DsPTP1*, At3g23610; *DREB2A*, At5g05410; *RD22*, At5g25610; *ICK1*, At2g23430; *ABF1*, At1g49720; *COR15A*, At2g42540; *ERD1*, At5g51070; *ZEP,* At5G67030; *NCED3*, At3g14440; *AAO3*, At2G27150; *CYP707A3*, At5G45340; *CYP707A4*, At3g19270; *ABI1*, At4g26080; *ABI3*, At3g24650; *ABI4*, At2g40220; and *ABI5*, At2g36270.

## Results

### Phenotypic analyses of the *dsptp1* mutant

**Fig. 1.**
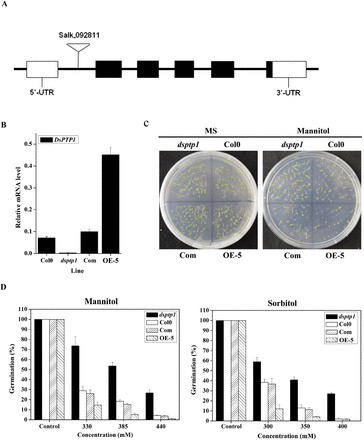
The *dsptp1* mutant shows an increased germination percentage under osmotic stress. (A) Structure of the *AtDsPTP1* gene. The insertion of T-DNA in the *dsptp1* mutant is shown as a triangle. Exons are shown as black boxes and introns as thin lines. (B) Real-time RT-PCR analysis for *AtDsPTP1* transcript. The *AtDsPTP1* transcript was present in total RNA from the WT, Com, and OE-5 lines but absent in the *dsptp1*-mutant plants. Relative expression levels of *AtDsPTP1* are normalized to *AtACTIN2* transcript levels. Values presented are mean ± SE of three different experiments. (C) Germination of WT (Col0), *dsptp1*-mutant, Com, and OE-5 line *Arabidopsis* seeds in normal and mannitol-containing media. (D) Percentage seed germination of *dsptp1-*mutant plants with and without different osmotic treatments. *Arabidopsis* seeds of WT (Col0) and *DsPTP1* mutants were sown in MS medium with or without osmotic treatments including 330, 385, and 440mM mannitol (left) and 300, 350, and 400mM sorbitol. Values presented are mean ± SE of three different experiments.

**Fig. 2.**
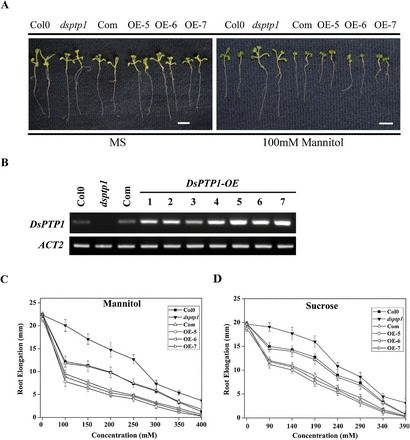
Root elongation in *dsptp1* mutants is insensitive to osmotic stress. (A) *Arabidopsis* root growth phenotypes of WT (Col0), *dsptp1*, and OE lines. Bar, 1cm. (B) The *AtDsPTP1* transcript was present in total RNA from the WT, Com, and OE-1 to OE-7 plants, but absent in *dsptp1*-mutant plants. Relative expression levels of *AtDsPTP1* were normalized to *AtACT2* transcript levels. Values presented are mean ± SE of three different experiments. (C, D) *dsptp1* seedlings show reduced root elongation inhibition under osmotic stress. Root lengths of Col0, *dsptp1*, and OE-5 line seedlings grown for 7 d on MS plates with mannitol (C) and sucrose (D) were measured to quantify their sensitivities to mannitol or sucrose. Seedlings were grown in MS medium for 1 week and then transferred to MS medium with or without various concentrations of mannitol and sucrose. Root elongation (i.e. increase in length after transfer) was determined after 7 d in Col0, *dsptp1*, and OE lines. Error bars indicate SE (*n* = 15). The experiments were repeated at least three times with similar results. Graphs correspond to one representative experiment.

To identify additional osmotic signalling components during seed germination, we obtained *Arabidopsis* T-DNA insertion mutants for the genes encoding various protein phosphatases. We also performed screening to identify any mutation that could lead to alteration in resistance to osmotic stress during seed germination. As shown in Fig. 1, *dsptp1* shows enhanced tolerance to osmotic stress. The *dsptp1* mutant carries a T-DNA insertion in the At3g23610 of the *AtDsPTP1* gene. The results of real-time PCR and RT-PCR show that *dsptp1* is a loss-of-function mutant because the *AtDsPTP1* transcript level was hardly detected in the mutant, but it was obviously enhanced in the *DsPTP1-OE* line (OE-1, 2, 3, 4, 5, 6, 7) compared to the wild type, and it returned to a level similar to the wild type in the *DsPTP1-Com* line (Figs 1A, B; Fig. 2B). For this study we selected three independent transgenic lines (OE-5, 6, 7) that show high expression levels of *DsPTP1* compared with the wild type. Under normal growth conditions in MS medium, no difference in germination percentage was observed among the WT, *dsptp1* mutant, OE line (OE-5, 6, 7) and Com line seeds. However, the *dsptp1*-mutant seeds exhibited a significantly higher seed germination percentage than WT, while the OE line exhibited a lower seed germination percentage than WT (Figs. 1C, D; Supplementary Figure S1A, B). The Com line rescued the seed germination percentage to a level similar to that for WT seeds when compared with OE line seeds in MS medium supplemented with mannitol.

To determine the sensitivity of the *dsptp1* mutant to osmotic stress during seed germination, different concentrations of mannitol (330mM, 385mM, and 440mM) and sorbitol (300mM, 350mM, and 400mM) were supplied in the medium. The results show that the *dsptp1* mutant exhibited an obviously higher seed germination percentage than WT, whereas the OE line exhibited the lowest seed germination percentage with the increasing mannitol and sorbitol concentrations (Fig. 1D; Supplementary Figures S1B, C).

### Overexpression of DsPTP1 decreases osmotic tolerance in *Arabidopsis*

We generated the *DsPTP1-OE* lines and the *DsPTP1-Com* lines. The OE lines showed an increase in At3g23610 at the *AtDsPTP1* transcript level relative to the WT plants, and the Com line showed a similar transcript level of *AtDsPTP1* compared with the WT plants (Figs 1B, 2B). The seedlings of the OE lines, Com lines, *dsptp1* mutant, and WT (Col0) were grown under osmotic stress. Their primary root growth was measured. Root growth of *dsptp1*-mutant plants was hyposensitive to mannitol (Fig. 2A, C; Supplementary Figure S1A, B) and sucrose (Fig. 2D; Supplementary Figure S1C) compared with that of WT plants (Col0). There was a similar result with a low concentration of sucrose in MS, which removes the sucrose (Supplementary Figure S4). By contrast, the OE lines were relatively more sensitive to osmotic agents than the WT (Fig. 2A, C, D; Supplementary Figure S1A, B, C), and the Com line rescued the root elongation of *dsptp1* to a level similar to WT (Figs. 2A, C, D). These data indicate that *AtDsPTP1* decreases osmotic stress tolerance in *Arabidopsis*.

### Expression patterns of *AtDsPTP1*

**Fig. 3.**
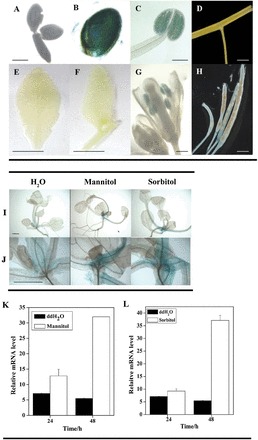
Expression patterns of the *DsPTP1* gene in seedlings. The *AtDsPTP1* promoter-GUS activity was observed at various growth stages (A–J). (A) Embryo of dry seed (A). (B) Seed coat. (C) Pollen in anther. (D) Stems. (E) Mature rosette leaves. (F) Cauline leaves. (G) Flowers. (H) Siliques. Six independent lines were tested and observations repeated at least three times. (I) Twelve-day-old seedling. (J) Enlargement of (I); the right-hand photo in (J) is turned by 90° from the photo in (I). Six independent lines were tested and repeated at least three times. (A, B, C) Bar, 20 µm. (E, F) bar, 1cm; (D, G, H) bar, 1mm; (I, J); bar, 1mm. (K, L) Time course of mannitol-induced (K) and sorbitol-induced (L) gene expression of *AtDsPTP1* in *Arabidopsis* seedlings. WT seedlings were treated with 440mM mannitol or 400mM sorbitol for various times. All seedlings treated with distilled water under the same conditions during the entire period served as controls. Relative expression levels of *AtDsPTP1* were analysed by qRT-PCR and normalized to *Atactin2* transcript levels. Values are presented are mean ± SE of three different experiments.

**Fig. 4.**
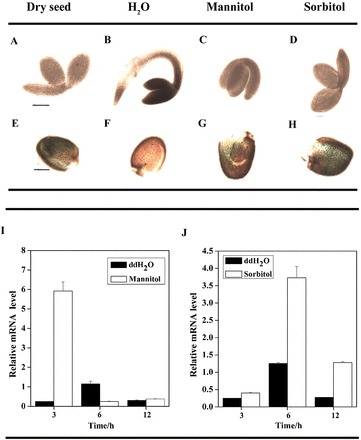
Induced expression patterns of the *DsPTP1* gene in seeds under osmotic stress. (A and E) Dry seed. (B–D). Three-day germinating seed. (F–H) Seed coat under different treatment conditions. Six independent lines were tested and repeated three times. Bar, 20 µm. (I, J) Time course of mannitol-induced (I) and sorbitol-induced (J) gene expression of *AtDsPTP1* in *Arabidopsis* seeds. The WT Col0 seeds were treated with 440mM mannitol (I) and 400mM sorbitol (J) for various times. In all cases, seeds treated with distilled water under the same conditions during the entire period served as controls. Relative expression levels of *AtDsPTP1* were analysed by qRT-PCR and normalized to *Atactin2* transcript levels. Values presented are mean ± SE of three different experiments.

We constructed a chimeric gene consisting of the *ATDsPTP1* promoter fused to a *GUS* reporter gene (*ATDsPTP1pro-GUS*) to analyse the expression patterns of GUS activity driven by the *ATDsPTP1* promoter in transgenic *Arabidopsis* plants. Spatiotemporal expression analysis showed GUS expression in seed endosperm (Fig. 3B), pollen grains (Fig. 3C, G), and abscission zones of siliques (Fig. 3I), but not in mature embryo (Fig. 3A), rosette, and cauline leaf (Fig. 3E, F). Strong GUS activity was detected in leaf petioles and roots of rosette plants when the transgenic plants were exposed to high osmolarity solutions (Fig. 3I, J). Similarly, strong GUS staining was observed in the seed coat (Fig. 4E, G, H), but not in the embryo (Figs. 4A, C, D) of dry and 3-d-germinating seeds that were exposed to both hyperosmotic solutions. These results suggest that the expression of the *AtDsPTP1* gene is upregulated in response to external osmotic changes.

We also assayed the transcript level of *DsPTP1* in germinating *Arabidopsis* seeds from 3 to 12h after imbibing and in 1 and 2 d seedlings under different treatments using qRT-PCR. In accordance with the results of GUS staining, the *AtDsPTP1* expression in WT seeds was sharply induced by mannitol (Fig. 4I) and sorbitol (Fig. 4J) solutions. The transcript level of *AtDsPTP1* in WT seedlings was also enhanced at 48h after mannitol (Fig. 3K) and sorbitol (Fig. 3L) treatments. These results indicate that *AtDsPTP1* is induced by osmotic stress.

### Increased proline content and resistance to oxidative damage in *dsptp1* plants

**Fig. 5.**
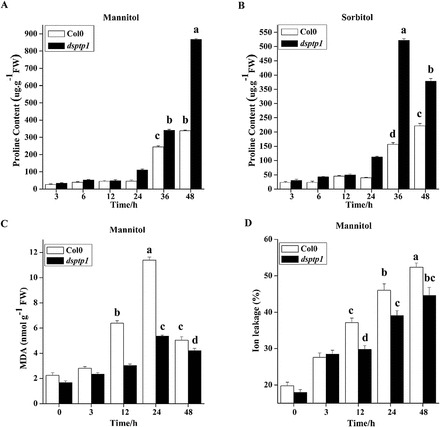
The *dsptp1* mutant affects the physiological index under osmotic stress. (A, B) Proline content determined in WT and *dsptp1* mutants under mannitol treatment (A) and sorbitol treatment (B). (C, D) MDA content (C) and ion leakage (D) determined in WT and *dsptp1* mutants under mannitol treatment. Values presented are mean ± SE of three different experiments. Means denoted by the same letter do not significantly differ at *P* < 0.05 according to Duncan’s multiple range test.

Proline accumulation has adaptive functions in plant stress tolerance, acting as a compatible osmolyte that stores carbon and nitrogen (Hare *et al.*, 1997). Previous studies *in vitro* showed that proline can be a reactive oxygen species (ROS) scavenger (Smirnoff and Cumbes, 1989), and a molecular chaperone that stabilizes protein structure. Proline accumulation can buffer cytosolic pH and balance cell redox status; it is also part of the stress signal that influences adaptive responses (Maggio *et al.*, 2002). Proline accumulation in the *dsptp1* mutant and WT plants treated with mannitol and sorbitol was measured. As shown in Fig. 5, the proline content in both *dsptp1* mutant and WT plants increased in response to 330mM mannitol treatment. However, the increase in the *dsptp1*-mutant plants was more remarkable than that in the WT plants at 48h after mannitol treatment (Fig. 5A). A similar trend in proline content was observed between the *dsptp1* mutant and WT plants treated with 400mM sorbitol for 36h (Fig. 5B).

MDA is produced by lipid peroxidation because of the effect of ROS (Halliwell and Chirico, 1993). As the best-characterized product of lipid peroxidation, MDA is often used as a biomarker to measure cell membrane injury and cell and tissue oxidative damage (Esterbauer *et al.*, 1991). To determine the degree of cellular injury, we determined the MDA content in the *dsptp1* mutant and WT plants with 550mM mannitol treatment. The MDA content increased in both *dsptp1*-mutant and WT plants after the treatment. However, the increase in the *dsptp1*-mutant seedlings was less than half of that in the WT seedlings after the treatment. Although the *dsptp1*-mutant seedlings had slightly lower MDA content than the WT seedlings even without stress treatment, the increase in the MDA level was significantly lower in the mutant than in the WT plants after 12 and 24h of mannitol treatment (Fig. 5C). Similar results were obtained with the 330mM mannitol treatment (Supplementary Figure S5).

After the *dsptp1*-mutant and WT seedlings were treated with 550mM mannitol, ion leakage was measured as an indicator of osmotic stress-induced cellular damage. Although the *dsptp1*-mutant seedlings had no obvious differences in ion leakage compared with the WT plants without stress treatment, mannitol treatment resulted in a sharper increase in electrolyte leakage in the WT plants than in the *dsptp1-*mutant plants (Fig. 5D). These data indicate that the *dsptp1*-mutant plants are less sensitive to osmotic stress.

**Fig. 6.**
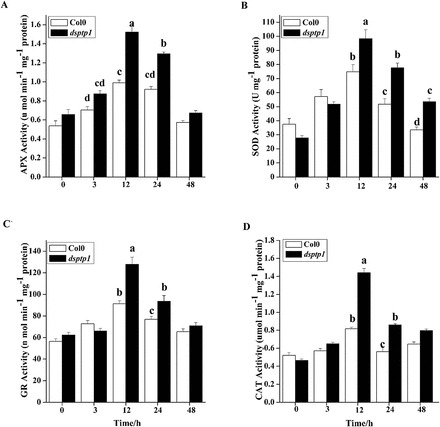
Effects of DsPTP1 on the activities of antioxidant enzymes in *Arabidopsis* seedlings exposed to mannitol treatment. (A–D) Total activities of antioxidant enzymes APX (A), SOD (B), GR (C), and CAT (D). Seedlings were treated with mannitol for the times indicated. Values are mean ± SE of three different experiments. Means denoted by the same letter do not significantly differ at *P* < 0.05 according to Duncan’s multiple range test.

To investigate the effects of AtDsPTP1 on the activities of antioxidant enzymes, we determined the total activities of antioxidant enzymes, including APX, SOD, GR, and CAT. Fig. 6 shows that the osmotic stress-induced APX (Fig. 6A), SOD (Fig. 6B), GR (Fig. 6C), and CAT (Fig. 6D) activities of the *dsptp1*-mutant plants are significantly higher than those of the WT plants. These findings suggest that *dsptp1* has an enhanced ability to scavenge the extra ROS under osmotic stress to maintain the equilibrium between ROS production and scavenging. These data indicate that the *dsptp1*-mutant plants can attenuate cell damage caused by osmotic stress.

### The *dsptp1* mutant enhances expression of osmotic responsive genes

**Fig. 7.**
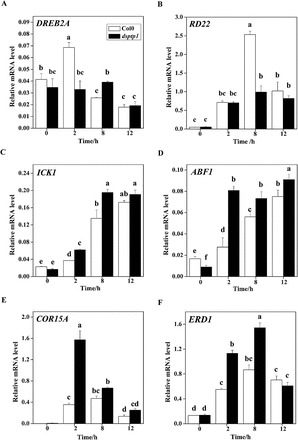
The *dsptp1* mutant shows altered expression of several dehydration-responsive genes after osmotic stress: *DREB2A* (A), *RD22* (B), *ICK* (C), *ABF* (D), *COR15A* (E), and *ERD1* (F). The expression level of these genes in response to stress was analysed by qRT-PCR. Total RNA was isolated from 15-d-old WT, Col0, and *dsptp1* seedlings treated with 385mM mannitol for 0h, 2h, 8h, and 12h. In all experiments, the expression of the constitutive *Actin2* gene was used as the control. Three replicates were made for each treatment with similar results. Values are mean ± SE of three different experiments. Means denoted by the same letter do not significantly differ at *P* < 0.05 according to Duncan’s multiple range test.

To determine the function of AtDsPTP1 in signalling pathways in response to osmotic stress, we analysed the expression of genes regulated by abiotic stress in WT and *dsptp1-*mutant plants with or without mannitol treatment. As shown in the real-time PCR results in Fig. 7, significant differences in the expression of general stress-responsive genes (e.g. *RD22* and *ICK1*) and ABA-response genes (e.g. *ABF1*) were found between the WT and the *dsptp1*-mutant plants. In addition, osmotic stress-induced expression was different in the *dsptp1*-mutant and WT seedlings for *ERD1* (early response to dehydration 1) (Fig. 7F), *COR15A* (Fig. 7E), and *DREB2A* (Fig. 7A). *ERD1* encodes a chloroplast-targeted chaperonin possibly involved in proteolysis (Weaver *et al.*, 1998), *COR15A* encodes a chloroplast-targeted LEA protein (Artus *et al.*, 1996), and *DREB2A* encodes a transcription factor activated in the early stages of the *ERD1* osmotic stress response (Kiyosue *et al.*, 1994).

Shortly after osmotic treatment, *DREB2A* and *RD22* transcripts accumulated to significantly higher levels in the WT than in the *dsptp1*-mutant plants (Figs. 7A, B). By contrast, the induction of *ICK1*, *ABF1*, *COR15A*, and *ERD1* expression was higher in the *dsptp1*-mutant plants than that in the WT plants after osmotic treatment (Figs 7C–F). The compromised induction of different dehydration-responsive genes together with reduced sensitivity to osmotic stress in *dsptp1* indicated that *DsPTP1* is possibly important in the dehydration-signalling pathway.

### The *dsptp1* mutant reduces ABA content and suppresses the ABA-signalling pathway under osmotic stress

**Fig. 8.**
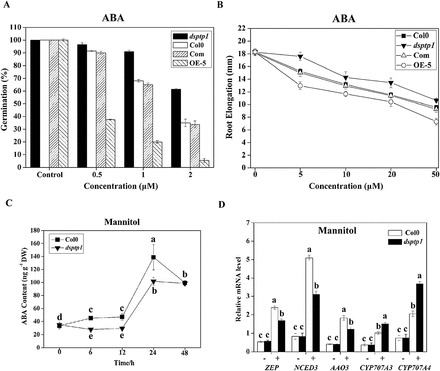
DsPTP1 is involved in osmotic stress- and ABA- signalling. (A) Percentage *Arabidopsis* seed germination with WT, *dsptp1*, OE, and Com lines in MS medium supplemented with or without 0.5, 1, and 2 μM ABA. (B) Seedlings were grown in MS medium for 1 week and then transferred to MS medium with or without 5, 10, 20, and 50 μM ABA. Root elongation (increase in length after transfer) was determined after 7 d. Error bars indicate SE (*n* = 15 for root elongation; *n* = 3 for germination; each experiment contained 100 seeds). Three replicates were made for each treatment with similar results, and graphs correspond to one representative experiment. (C) Determination of endogenous ABA level in WT and *dsptp1* seedlings after mannitol treatments. Expression differences of the ABA-biosynthesis key genes *ZEP*, *NCED*, *AAO3*, and catabolic gene *CYP707A3* and *CYP707A4*, in WT and *dsptp1* were analysed before or after osmotic stress. Values are mean ± SE of three different experiments. Means denoted by the same letter do not significantly differ at *P* < 0.05 according to Duncan’s multiple range test.

To test the sensitivity of the *dsptp1* mutant to ABA, we determined the effect of ABA on the germination, cotyledon expansion, and root elongation of *dsptp1*. The radicle emergence of seeds was observed after 7 d of sowing on MS medium supplemented with different ABA concentrations (Fig. 8A; Supplementary Figure S2A). The inhibition of seed germination by ABA in the *dsptp1*-mutant plants was less obvious than that in the WT plants, whereas the OE-line seeds were more sensitive to ABA than were the WT seeds. Seed germination percentage in the Com lines was similar to that of WT seeds. The effects of ABA on root elongation were also observed among *dsptp1*, WT, OE lines and Com lines. Root elongation was measured 7 d after 7-d-old seedlings were transferred to MS medium supplemented with different ABA concentration (Fig. 8B; Supplementary Figure S2B). The inhibition of root elongation by ABA was reduced in the *dsptp1*-mutant plants and enhanced by overexpression of DsPTP1 compared with the WT plants. Root elongation in the Com lines was similar to that of WT seeds (Fig. 8B).

The ABA contents in the *dsptp1*-mutant and WT plants were also measured using a radioimmunoassay (RIA) method. In the absence of stress treatment, WT and *dsptp1* leaves had similar ABA contents (Fig. 8C). When the plants were treated with 440mM mannitol for the indicated time, the endogenous ABA level in the WT plants was higher than that in the *dsptp1*-mutant plants.

To investigate the function of *DsPTP1* in ABA accumulation in response to osmotic stress, we analysed the expression of genes encoding enzymes for ABA biosynthesis and catabolism in the WT and *dsptp1*-mutant plants (Nambara and Marion-Poll, 2005). The qRT-PCR results showed that the expressions of all the ABA-biosynthesis genes and the catabolic genes are similar without osmotic stress. The expression level of all the genes assayed for ABA biosynthesis (*ZEP*, *NCED3*, and *AAO3*) and catabolism (*CYP707A3* and *CYP707A4*) increased after osmotic stress treatment. However, the magnitudes of the increases varied. The expression level of the biosynthesis gene *NCED3* markedly increased to a higher level in the WT plants compared with the *dsptp1*-mutant plants (Fig. 7D). By contrast, the catabolic gene *CYP707A4* was expressed at an obviously lower level in the WT plantscompared with the *dsptp1* mutant (Fig. 7D). This result indicates that DsPTP1 affects the endogenous ABA content by regulating the genes for ABA biosynthesis and catabolism after osmotic stress treatment.

**Fig. 9.**
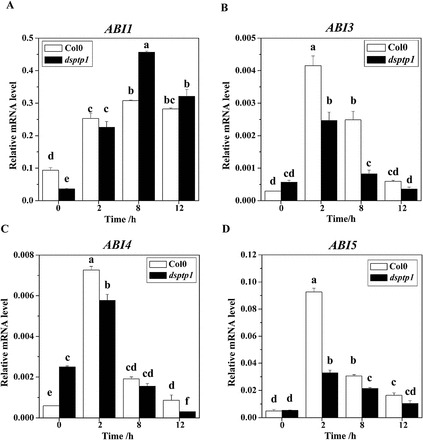
The *dsptp1* mutant shows altered expression of ABA-regulated genes under osmotic stress. Expression levels of several genes in response to ABA signalling were analysed by qRT-PCR (A–D): *ABI1* (A), *ABI3* (B), *ABI4* (C), and *ABI5* (D). Total RNA was isolated from 15-d-old WT and *dsptp1* seedlings treated with 385mM mannitol for the times indicated. In all experiments, the expression of the constitutive *Actin2* gene was used as the control. Three replicates were made for each treatment with similar results. Values are mean ± SE of three different experiments. Means denoted by the same letter do not significantly differ at *P* < 0.05 according to Duncan’s multiple range test.

To explore the function of AtDsPTP1 in ABA-signalling pathways in response to osmotic stress, we analysed the expression of genes that regulate ABA responses in WT and *dsptp1*-mutant plants both before and after mannitol treatment. The results of real-time PCR showed significant differences between the WT and the *dsptp1*-mutant plants in terms of the expression of ABA-regulated genes, such as *ABI1*, *ABI3*, and *ABI5* (Fig. 9). This indicates that *AtDsPTP1* probably acts upstream of these genes in the ABA pathways. The induction of *ABI3* (Fig. 9B) and *ABI5* (Fig. 9D) expression was significantly lower in *dsptp1*-mutant plants than in the WT plants with mannitol treatment. By contrast, the induction of *ABI1* increased in the *dsptp1*-mutant plants compared with the WT plants after osmotic stress (Fig. 9A). We also measured the expression of ABA-response genes (*ABI1*, *3*, *4*, *5*) in the WT and *dsptp1* mutant under 20 µM ABA treatment; the *dsptp1* mutant showed less sensitivity to ABA compared with the WT (Supplementary Figure S3).

## Discussion

Protein kinases and PPs have critical functions in plant development and stress responses (Fujii and Zhu, 2012). Members from different sub-families of PPs were studied for their biological functions (Schweighofer *et al.*, 2004; Schweighofer and Meskiene, 2008; Bartels *et al.*, 2010). As a member of the unique group that is capable of dephosphorylating both serine/threonine and tyrosine, DsPTP1 is little known, especially in plant stress responses. Thus, we characterized a *dsptp1* mutant that is hyposensitive to osmotic stress during seed germination and root elongation of seedlings (Figs 1, 2; Supplementary Figure S1). With the genetic evidence, we studied the biological function of the *DsPTP1* gene in response to stress conditions. The expression of the *DsPTP1* gene is induced by osmotic stresses both in seedlings (Fig. 3K, L) and seeds (Fig. 4I, J). Knockout of the *AtDsPTP1* gene results in enhanced tolerance, whereas overexpression of the *AtDsPTP1* gene results in an attenuated tolerance compared with the wild type, and the *AtDsPTP1*-complemented lines rescued the tolerance of the *dsptp1* mutant to the wild-type level under these stress conditions (Figs 1, 2; Supplementary Figure S1). Our results indicate that the *AtDsPTP1* gene is involved in osmotic stress responses and possibly has a negative regulatory function.

A promoter-reported fusion analysis indicated that *DsPTP1* is strongly expressed in the endosperm layer in the seed coat of dry seeds (Figs 3B, 4E), but that this disappears after imbibition (Fig. 4F). Seed coats have a key function in maintaining seed dormancy (Holdsworth *et al.*, 2008), suggesting that *DsPTP1* is involved in regulating seed germination; the induced expression of *DsPTP1* occurs at the base and roots of rosettes, both of which are important for the maintenance of root elongation under stress conditions (Beemster and Baskin, 1998). These results further prove that *AtDsPTP1* is a negative regulator in plant growth and development under stress conditions, such as seed germination and root elongation.

Although most protein phosphatases have many functions in controlling plant growth and development, the pathways they are involved in can vary. We studied the physiological and biochemical responses of the *dsptp1* mutant to osmotic stress. As shown in Fig. 5, all physiological indexes, such as ion leakage, suggest a better performance of the *dsptp1* mutant under osmotic stress than WT. Proline accumulates largely in cells as an osmolyte to help in osmotic adjustment under stress. The *dsptp1*-mutant plants had higher proline accumulation than the WT plants under osmotic stress. Proline accumulation modulates pH changes and reduces cell damage by osmotic stress (Zhu, 2002). The levels of MDA, a biomarker of cell membrane injury, and ion leakage, an indicator of osmotic-induced cellular damage, increase less under osmotic stress in the *dsptp1* mutant than in WT.

ROS are important signalling molecules that accumulate under many stress conditions. However, high ROS concentrations can be highly toxic to plants (Apel and Hirt, 2004; Ye *et al.*, 2011). Thus, we measured the antioxidant enzyme activities in the *dsptp1*-mutant and WT plants (Fig. 6). Increased proline content reduces the inhibitory effects of osmotic stress on enzyme activity, increases the thermal stability of enzymes, and prevents the dissociation of enzyme complexes (Hayashi *et al.*, 1997). Therefore, we suspect that proline accumulation causes the enhanced activity of all four antioxidant enzymes in the *dsptp1* mutant relative to the WT plants, which could explain why the MDA content in the mutant plant is lower than that in the WT plants.

The results demonstrate that *DsPTP1* is induced by osmotic stress in the WT plants, thereby suppressing the accumulation of many metabolites that ameliorate the effects of osmotic stress and changing the expression pattern of a subset of dehydration-responsive genes (Fig. 7). Knockout of the *AtDsPTP1* gene in the *dsptp1-*mutant plants results in better adaption to osmotic stress than in the WT plants. The compromised induction of different dehydration-responsive genes together with reduced sensitivity to osmotic stress in *dsptp1* indicates that *DsPTP1* is possibly important in the dehydration signalling pathway (Fig. 7).

As a stress phytohormone, ABA has a pivotal regulatory function in numerous stress responses, including osmotic stress (Boudsocq and Lauriere, 2005; Rosado *et al.*, 2006; Cutler *et al.*, 2010). To study how the *DsPTP1* gene controls the responses of plant to osmotic stress, we examined the relationship between the *DsPTP1* gene and ABA using the *dsptp1*-mutant plants. The inhibitory effects of ABA on seed germination and root elongation were suppressed by the mutation of the *AtDsPTP1* gene but enhanced by the overexpression of the *AtDsPTP1* gene (Fig. 8A, B; Supplementary Figs S2A, B). Meanwhile, the seed germination percentage (Fig. 8A) and root elongation (Fig. 8B) were rescued to a level similar to the wild type by complementing the corresponding gene in the *dsptp1* mutant. Similar effects were noted under osmotic stress treatment (Figs 1, 2). These results strongly suggest that the *AtDsPTP1* gene is involved in the *Arabidopsis* ABA-signalling pathway, mediating the inhibitory effect of ABA in plants under osmotic stress.

To study the relationship between AtDsPTP1 and the ABA pathway, we determined the ABA contents in the *dsptp1* mutant and WT plants. Knockout of the *DsPTP1* gene decreased the ABA content in the *dsptp1*-mutant plants compared with the WT plants. This finding may be attributed to the downregulated expression of the rate-limiting, ABA-biosynthesis enzyme gene *NCED3*, and the significantly upregulated expression of the ABA-catabolic enzyme gene *CYP707A4* (Fig. 8).

Thus, downregulation of AtDsPTP1 can decrease ABA content by suppressing ABA biosynthesis and increasing ABA catabolism. The expression of genes in the ABA-response pathway show similar results (Fig. 9); downregulation of DsPTP1 decreases the gene expression of the positive regulators in ABA signalling, such as ABI3 and ABI5, and increases the expression of the negative regulator, ABI1, under osmotic stress conditions. This phenomenon can also explain why the mutant plant is less sensitive to osmotic stress as well as the ABA treatment. These findings suggest that the *dsptp1*-mutant plants are more tolerant to osmotic stress than the WT plants.

All of these results show that tolerance of *dsptp1* to osmotic stress is mainly connected to proline accumulation. With a higher concentration of proline accumulation in *dsptp1*, there is less injury under osmotic stress. Thus, the *dsptp1* mutant accumulated less ABA, which led to a decreased ABA response compared with the WT under osmotic treatment (Fig. 8C, D, Fig. 9; Supplementary Figure S5), just as previously reported (Lv *et al.*, 2011). The *dsptp1* mutant also showed less sensitivity to exogenous ABA, and this is also connected with the lower accumulation of, and decreased the sensitivity to, ABA (Fig. 8A, B; Supplementary Figure S2). These results show that DsPTP1 acts as a negative regulator in osmotic stress signalling during *Arabidopsis* seed germination and seedling establishment.

We characterized a *dsptp1* mutant of *Arabidopsis*. The *DsPTP1* gene is induced significantly by osmotic stress and has a negative regulatory function during seed germination and seedling establishment. Its mutation results in better performance in the *dsptp1*-mutant than WT plants under osmotic stress. The *dsptp1*-mutant plants accumulated more proline than WT plants under osmotic stress; proline functions in osmotic adjustment in cells. The *dsptp1*-mutant plants exhibit attenuated sensitivity to osmotic stress damage than the WT plants, resulting in reduced MDA content, reduced ion leakage, and enhanced antioxidant enzyme activities (APX, SOD, GR, and CAT). The accumulation of ABA in response to osmotic stress is also controlled by downregulation of *DsPTP1* gene expression, which reduces expression of ABA-biosynthesis genes and increases expression of ABA-catabolism genes. The *DsPTP1* gene is also involved in the ABA-signalling pathway, regulating the expression of ABI genes such as *ABI1*, *ABI3*, and *ABI5*. In addition, DsPTP1 regulates the expression of a series of dehydration-responsive genes under osmotic stress. The aforementioned results show that DsPTP1 acts as a negative regulator in osmotic stress signalling during *Arabidopsis* seed germination and seedling establishment.

## Supplementary material

Supplementary data can be found at *JXB* online.

Supplementary Table S1. Primers used for real- time RT-PCR assays.

Supplementary Figure S1. Comparison of WT, *dsptp1*-mutant, OE-6, and OE-7 lines in seed germination under osmotic stresses.

Supplementary Figure S2. Comparison of WT, *dsptp1*-mutant, OE-6, and OE-7 lines in seed germination and root elongation under ABA treatment.

Supplementary Figure S3. The *dsptp1* mutant shows altered expressions of ABA-regulated genes under ABA treatment.

Supplementary Figure S4. Comparison of root elongation in the OE, WT, and *dsptp1*-mutant lines on medium with or without sucrose.

Supplementary Figure S5. Comparison of MDA content between the WT and *dsptp1* mutant under treatment with 330mM mannitol.

Supplementary Data

## References

[CIT0001] Agarwal PK Jha B 2010 Transcription factors in plants and ABA dependent and independent abiotic stress signalling. Plant Biology 54, 201–212.

[CIT0002] Apel K Hirt H 2004 Reactive oxygen species: metabolism, oxidative stress, and signal transduction. Annual Review of Plant Biology 55, 373–399. 10.1146/annurev.arplant.55.031903.141701 15377225

[CIT0003] Artus NN Uemura M Steponkus PL Gilmour SJ Lin C Thomashow MF 1996 Constitutive expression of the cold-regulated *Arabidopsis* thaliana COR15a gene affects both chloroplast and protoplast freezing tolerance. Proceedings of the National Academic of Sciences, USA 93, 13404–13409. 10.1073/pnas.93.23.13404 PMC24106 11038526

[CIT0004] Bartels S Besteiro MA Lang D Ulm R 2010 Emerging functions for plant MAP kinase phosphatases. Trends in Plant Science 15, 322–329. 20452268 10.1016/j.tplants.2010.04.003

[CIT0005] Bates LS 1973 Rapid determination of free proline for water-stress studies. Plant and Soil 39, 205–207.

[CIT0006] Bechtold N Ellis J Pelletier G 1993 In planta Agrobacterium-mediated gene transfer by infiltration of adult *Arabidopsis thaliana* plants. Comptes Rendus de l Academie des Sciences Series III 316, 1194–1199.

[CIT0007] Beemster GTS Baskin TI 1998 Analysis of cell division and elongation underlying the developmental acceleration of root growth in *Arabidopsis thaliana* . Plant Physiology 116, 1515–1526. 9536070 10.1104/pp.116.4.1515 PMC35060

[CIT0008] Boudsocq M Lauriere C 2005 Osmotic signaling in plants: multiple pathways mediated by emerging kinase families. Plant Physiology 138, 1185–1194. 16009994 10.1104/pp.105.061275 PMC1176393

[CIT0009] Bradford MM 1976 A rapid and sensitive method for the quantitation of microgram quantities of protein utilizing the principle of protein-dye binding. Annals of Biochemistry 72, 248–254. 10.1016/0003-2697(76)90527-3 942051

[CIT0010] Clough SJ Bent AF 1998 Floral dip: a simplified method for Agrobacterium-mediated transformation of Arabidopsis thaliana. The Plant Journal 16, 735–743. 10069079 10.1046/j.1365-313x.1998.00343.x

[CIT0011] Cutler SR Rodriguez PL Finkelstein RR Abrams SR 2010 Abscisic acid: Emergence of a core signaling network. Annual Review of Plant Biology 61, 651–679. 10.1146/annurev-arplant-042809-112122 20192755

[CIT0012] Droillard MJ Boudsocq M Barbier-Brygoo H Lauriere C 2004 Involvement of MPK4 in osmotic stress response pathways in cell suspensions and plantlets of *Arabidopsis thaliana*: activation by hypoosmolarity and negative role in hyperosmolarity tolerance. FEBS Letters 574, 42–48. 15358537 10.1016/j.febslet.2004.08.001

[CIT0013] Esterbauer H Schaur RJ Zollner H 1991 Chemistry and biochemistry of 4-hydroxynonenal, malonaldehyde and related aldehydes. Free Radical Biology and Medicine 11(1 **),** 81–128. 1937131 10.1016/0891-5849(91)90192-6

[CIT0014] Fujita Y Fujita M Shinozaki K Yamaguchi-Shinozaki K 2011 ABA-mediated transcriptional regulation in response to osmotic stress in plants. Journal of Plant Research 124, 509–525. 21416314 10.1007/s10265-011-0412-3

[CIT0015] Fujii H Zhu JK 2012 Osmotic stress signaling via protein kinases. Cellular and Molecular Life Sciences 69, 3165–3173. 22828864 10.1007/s00018-012-1087-1 PMC3438365

[CIT0016] Gilmour SJ Fowler SG Thomashow MF 2004 Arabidopsis transcriptional activators CBF1, CBF2, and CBF3 have matching functional activities. Plant Molecular Biology 54, 767–781. 15356394 10.1023/B:PLAN.0000040902.06881.d4

[CIT0017] Gupta R Huang Y Kieber J Luan S 1998 Identification of a dual-specificity protein phosphatase that inactivates a MAP kinase from Arabidopsis. The Plant Journal 16, 581–589. 10036776 10.1046/j.1365-313x.1998.00327.x

[CIT0018] Gupta R Luan S 2003 Redox regulation of protein tyrosine phosphatases and MAP kinases in higher plants. Plant Physiology 132, 1149–1152. 12857797 10.1104/pp.103.020792 PMC1540326

[CIT0019] Gupta R Ting JT Sokolov LN Johnson SA Luan S 2002 A tumor suppressor homolog, AtPTEN1, is essential for pollen development in Arabidopsis. The Plant Cell 14, 2495–2507. 12368500 10.1105/tpc.005702 PMC151231

[CIT0020] Halliwell B Chirico S 1993 Lipid peroxidation: its mechanism, measurement, and significance. The American Journal of Clinical Nutrition 57, 715S–725S. 8475889 10.1093/ajcn/57.5.715S

[CIT0021] Hare PD Cress WA Van Staden J 1997 The involvement of cytokinins in plant responses to environmental stress. Plant Growth Regulation 23, 79–103.

[CIT0022] Hasegawa PM Bressan RA Zhu JK Bohnert HJ 2000 Plant cellular and molecular responses to high salinity. Annual Review of Plant Physiology and Plant Molecular Biology 51, 463–499. 10.1146/annurev.arplant.51.1.463 15012199

[CIT0023] Hayashi H Mustardy L Deshnium P Ida M Murata N 1997 Transformation of *Arabidopsis thaliana* with the coda gene for choline oxidase; accumulation of glycinebetaine and enhanced tolerance to salt and cold stress. The Plant Journal 12, 133–142. 9263456 10.1046/j.1365-313x.1997.12010133.x

[CIT0024] Heath RL Packer L 1968 Photoperoxidation in isolated chloroplasts. I. Kinetics and stoichiometry of fatty acid peroxidation. Archives of Biochemistry and Biophysics 125, 189–198. 5655425 10.1016/0003-9861(68)90654-1

[CIT0025] Holdsworth MJ Finch-Savage WE Grappin P Job D 2008 Postgenomics dissection of seed dormancy and germination. Trends in Plant Science 13, 7–13. 18160329 10.1016/j.tplants.2007.11.002

[CIT0026] Huang YF Li H Gupta R Morris PC Luan S Kieber JJ 2000 ATMPK4, an Arabidopsis homolog of mitogen-activated protein kinase, is activated in vitro by AtMEK1 through threonine phosphorylation. Plant Physiology 122, 1301–1310. 10759527 10.1104/pp.122.4.1301 PMC58966

[CIT0027] Jiang M Zhang J 2001 Effect of abscisic acid on active oxygen species, antioxidative defence system and oxidative damage in leaves of maize seedlings. Plant and Cell Physiology 42, 1265–1273. 11726712 10.1093/pcp/pce162

[CIT0028] Keyse SM 1995 An emerging family of dual specificity MAP kinase phosphatases. Biochemica et Biophysica Acta 1265, 152–160. 10.1016/0167-4889(94)00211-v 7696343

[CIT0029] Kiyosue T Yamaguchi-Shinozaki K Shinozaki K 1994 Cloning of cDNAs for genes that are early-responsive to dehydration stress (ERDs) in *Arabidopsis thaliana*: identification of three ERDs as HSP cognate genes. Plant Molecular Biology 25, 791–798. 8075396 10.1007/BF00028874

[CIT0030] Lv WT Lin B Zhang M Hua XJ 2011 Proline accumulation is inhibitory to Arabidopsis seedlings during heat stress. Plant Physiology 156, 1921–1933. 21670222 10.1104/pp.111.175810 PMC3149957

[CIT0031] Maggio A Matsumoto T Hasegawa PM Pardo JM Bressan RA 2002 The long and winding road to halotolerance genes. In: Lauchli A Luttge U, eds. Salinity: environment–plants–molecules. The Netherlands: Kluwer Academic Publishers, 505–533.

[CIT0032] Meskiene I Baudouin E Schweighofer A Liwosz A Jonak C Rodriguez PL Jelinek H Hirt H 2003 Stress-induced protein phosphatase 2C is a negative regulator of a mitogen-activated protein kinase. The Biochemical Journal 278, 18945–18952. 10.1074/jbc.M300878200 12646559

[CIT0033] Meskiene I Bogre L Glaser W Balog J Brandstotter M Zwerger K Ammerer G Hirt H 1998 MP2C, a plant protein phosphatase2C, functions as a negative regulator of mitogen-activated protein kinase pathways in yeast and plants. Proceedlings of the National Academy of Sciences, USA 95, 1938–1943. 10.1073/pnas.95.4.1938 PMC19217 9465121

[CIT0034] Murashige T Skoog F 1962 A revised medium for rapid growth and bioassays with tobacco tissue cultures. Physiologia Plantarum 15, 473–497.

[CIT0035] Nambara E Marion-Poll A 2005 Abscisic acid biosynthesis and catabolism. Annual Review of Plant Biology 56, 165–185. 10.1146/annurev.arplant.56.032604.144046 15862093

[CIT0036] Naoi K Hashimoto T 2004 A semidominant mutation in an *Arabidopsis* mitogen-activated protein kinase phosphatase-like gene compromises cortical microtubule organization. The Plant Cell 16, 1841–1853. 15208393 10.1105/tpc.021865 PMC514165

[CIT0037] Quarrie SA Whitford PN Appleford NEJ 1988 A monoclonal antibody to (S)-abscisic acid: its characterization and use in a radioimmunoassay for measuring abscisic acid in crude extracts of cereal and lupin leaves. Planta 173, 330–339. 24226540 10.1007/BF00401020

[CIT0038] Rosado A Amaya I Valpuesta V Cuartero J Botella MA Borsani O 2006 ABA and ethylene-mediated responses in osmotically stressed tomato are regulated by the TSS2 and TOS1 loci. Journal of Expermental Botany 57, 3327–3335. 10.1093/jxb/erl094 16914505

[CIT0039] Schweighofer A Hirt H Meskiene I 2004 Plant PP2C phosphatases: emerging functions in stress signaling. Trends in Plant Science 9, 236–243. 15130549 10.1016/j.tplants.2004.03.007

[CIT0040] Schweighofer A Meskiene I 2008 Protein phosphatases in plant growth signalling pathways. In: Boegre L, ed. Plant growth signalling. Heidelberg: Springer-Verlag, 277–297.

[CIT0041] Smirnoff N Cumbes QJ 1989 Hydroxyl radical scavenging activity of compatible solutes. Phytochemistry 28, 1057–1060.

[CIT0042] Ulm R Ichimura K Mizoguchi T Peck SC Zhu T Wang X Shinozaki K Paszkowski J 2002 Distinct regulation of salinity and genotoxic stress responses by Arabidopsis MAP kinase phosphatase 1. The EMBO Journal 21, 6483–6493. 12456655 10.1093/emboj/cdf646 PMC136950

[CIT0043] Ulm R Revenkova E di Sansebastiano GP Bechtold N Paszkowski J 2001 Mitogenactivated protein kinase phosphatase is required for genotoxic stress relief in Arabidopsis. Genes and Development 15, 699–709. 11274055 10.1101/gad.192601 PMC312655

[CIT0044] Weaver LM Gan S Quirino B Amasino RM 1998 A comparison of the expression patterns of several senescence-associated genes in response to stress and hormone treatments. Plant Molecular Biology 37, 455–469. 9617813 10.1023/a:1005934428906

[CIT0045] Xiong L Schumaker K Zhu JK 2002 Cell signaling during cold, drought and salt stress. The Plant Cell 14, S165–S183. 12045276 10.1105/tpc.000596 PMC151254

[CIT0046] Xiong L Zhu JK 2002 Molecular and genetic aspects of plant responses to osmotic stress. Plant, Cell and Environment 25, 131–139. 10.1046/j.1365-3040.2002.00782.x 11841658

[CIT0047] Ye N Jia L Zhang J 2012 ABA signal in rice under stress conditions. Rice 10, 1186/1939-8433-5-1. 10.1186/1939-8433-5-1 PMC3834477 24764501

[CIT0048] Ye N Zhu G Liu Y Li Y Zhang J 2011 ABA control H_2_O_2_ accumulation through the induction of *OsCATB* in Rice leaves under water stress. Plant and Cell Physiology 52, 689–698. 21398647 10.1093/pcp/pcr028

[CIT0049] Zhu JK 2002 Salt and drought stress signal transduction in plants. Annual Review of Plant Biology 53, 247–273. 10.1146/annurev.arplant.53.091401.143329 PMC3128348 12221975

