# Supplementary material for: AtDsPTP1 acts as a negative regulator in osmotic stress signalling during Arabidopsis seed germination and seedling establishment
Source: J Exp Bot. 2014 Dec 24;66(5):1339–53. doi: 10.1093/jxb/eru484 (PMC4339596; doi:10.1093/jxb/eru484)
Supplement: Supplementary Data [file supp_66_5_1339__index.html]

AtDsPTP1 acts as a negative regulator in osmotic stress signalling during Arabidopsis seed germination and seedling establishment — AtDsPTP1 acts as a negative regulator in osmotic stress signalling during Arabidopsis seed germination and seedling establishment — Supplementary Data 

# AtDsPTP1 acts as a negative regulator in osmotic stress signalling during *Arabidopsis* seed germination and seedling establishment

## Supplementary Data

Data files

**Files in this Data Supplement:**

- Supplementary Data - Supplementary Data
