# Supplementary material for: AtDsPTP1 acts as a negative regulator in osmotic stress signalling during Arabidopsis seed germination and seedling establishment
Source: J Exp Bot. 2014 Dec 24;66(5):1339–53. doi: 10.1093/jxb/eru484 (PMC4339596; doi:10.1093/jxb/eru484)
Supplement: Supplementary Data [file supp_eru484_jexbot129361_file001.pdf]

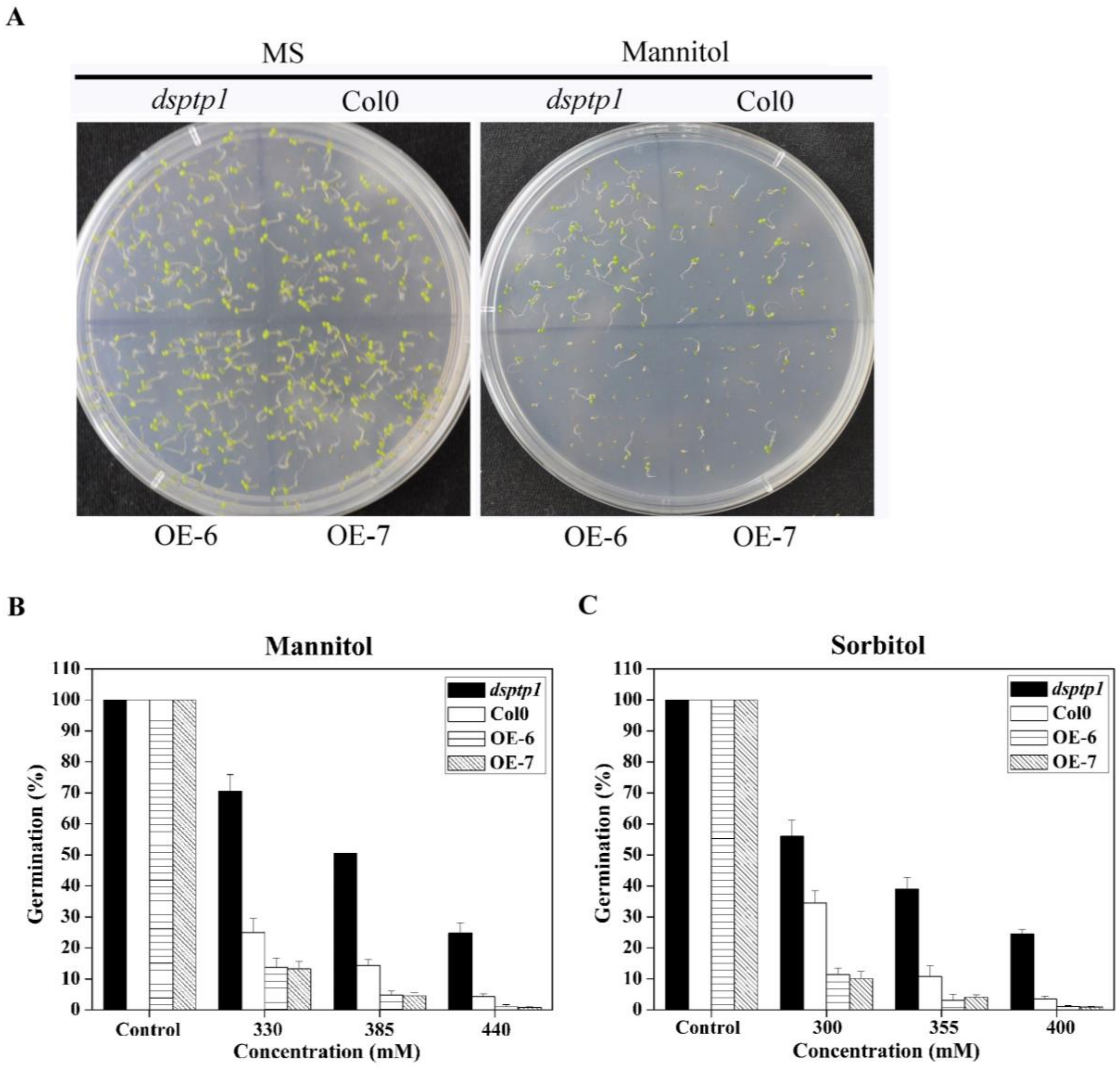

Supplementary Fig. S1. Comparison among WT, the *dsptp1* mutant, OE-6 and OE-7 lines in seed germination under osmotic stresses.

Germination of WT (Col0), the *dsptp1* mutant, and the independent Overexpression line (OE-6 and OE-7) *Arabidopsis* seeds in normal and mannitol-containing media (A). Seed germination percentage of the Col0, the *dsptp1* mutant, OE-6 and OE-7 line plants seeds with and without different osmotic treatments are shown (B, C). *Arabidopsis* seeds of WT (Col0) and *DsPTP1* mutant, OE-6 and OE-7 line are sown in MS medium with or without osmotic treatments including 330, 385, and 440mM mannitol (B) as well as 300, 350, and 400mM sorbitol (C). Values are presented means ± SE of three different experiments.

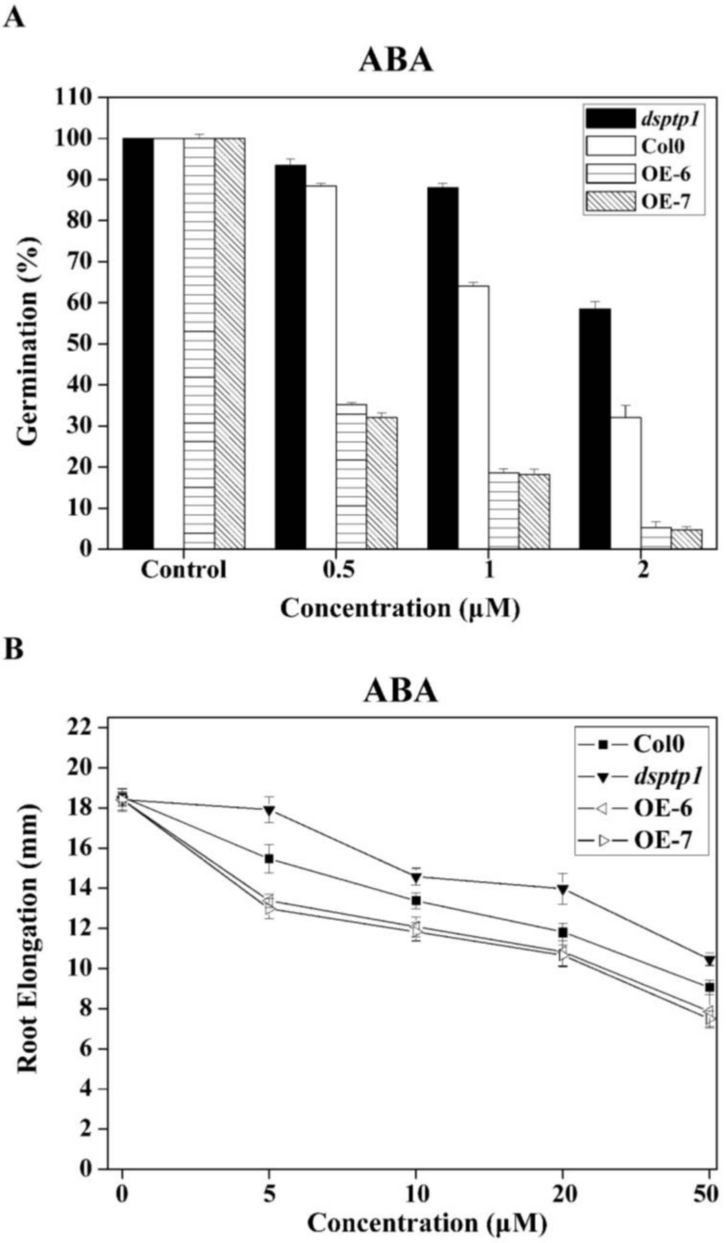

Supplementary Fig. S2. Comparison among WT, the *dsptp1* mutant, OE-6 and OE-7 lines in seed germination and root elongation under ABA treatment.

Seed germination percentage of *Arabidopsis* seeds of WT, *dsptp1*, independent OE (OE-6 and OE-7) lines in MS medium supplemented with or without 0.5, 1, and 2 μM ABA (A). Seedlings were grown in MS agar medium for 1 week and then transferred to MS medium with or without 5, 10, 20, and 50 μM ABA (B). Root elongation (increase in length after transfer) was determined after 7d. Error bars indicate SEs (n=15 for root elongation, n=3 for germination, each experiment contains 100 seeds). Three replicates were made for each treatment with similar results, and graphs correspond to one representative experiment.

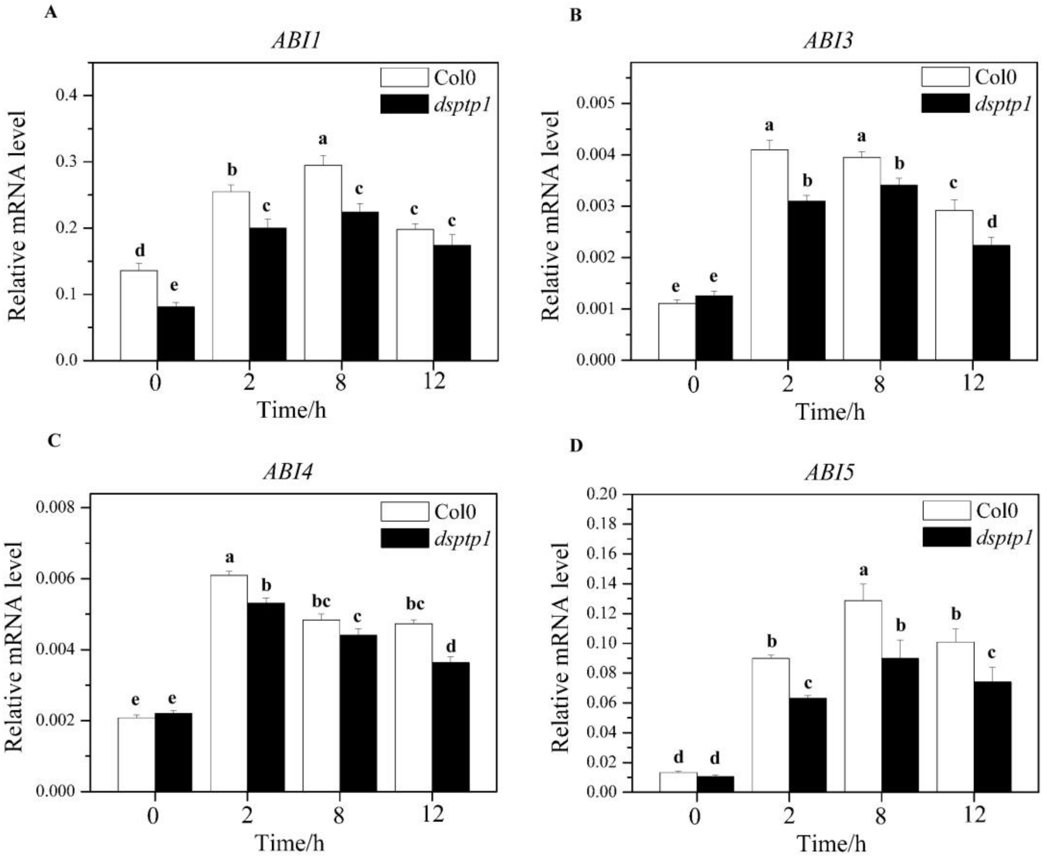

Supplementary Fig. S3. *dsptp1* shows altered expressions of ABA-regulated genes under ABA treatment. Expression level of several genes in response to ABA signaling was analyzed by qRT-PCR (A-D). *ABI1* (A) *ABI3* (B) *ABI4* (C) and *ABI5* (D). Total RNA was isolated from 15-day-old WT and *dsptp1* seedlings treated with 20 μM ABA for indicated times. In all experiments, the expression of the constitutive *Actin2* gene was used as the control. Three replicates were made for each treatment with similar results. Values are means ± SE of three different experiments. Means denoted by the same letter do not significantly differ at $P < 0.05$ according to Duncan's multiple range test.

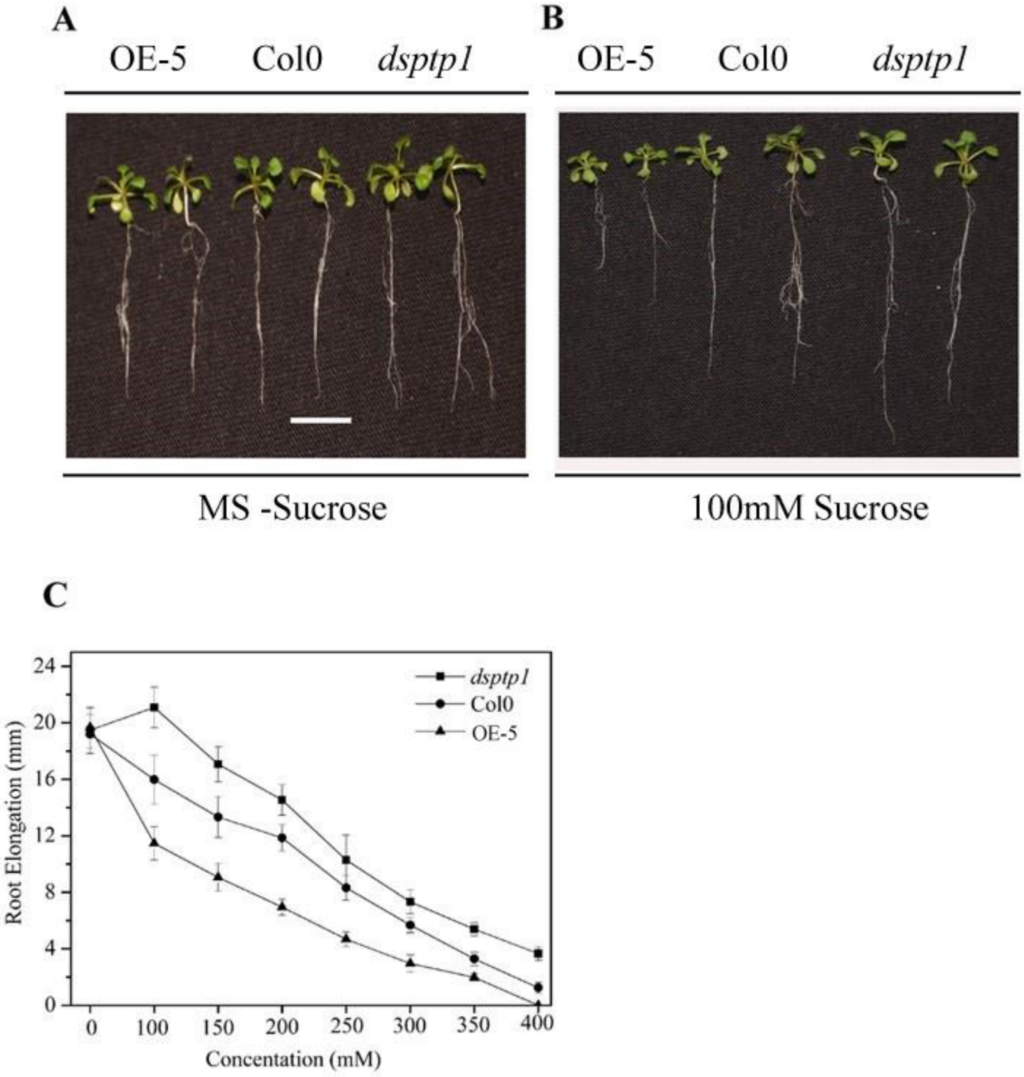

Supplementary Fig. S4. Comparison of root elongation among the OE, WT and *dsptp1* mutant on the MS-Sucrose and MS supplemented with 100mM Sucrose.

Seedlings were grown in MS agar medium for 1 week and then transferred to basic MS medium without sucrose (A, C) and MS medium supplemented with 100mM sucrose (B, C). The bar = 1cm. Root elongation (i.e. increase in length after transfer) was determined after 7d in Col0, *dsptp1* and OE. The experiments were repeated at least thrice with similar results.

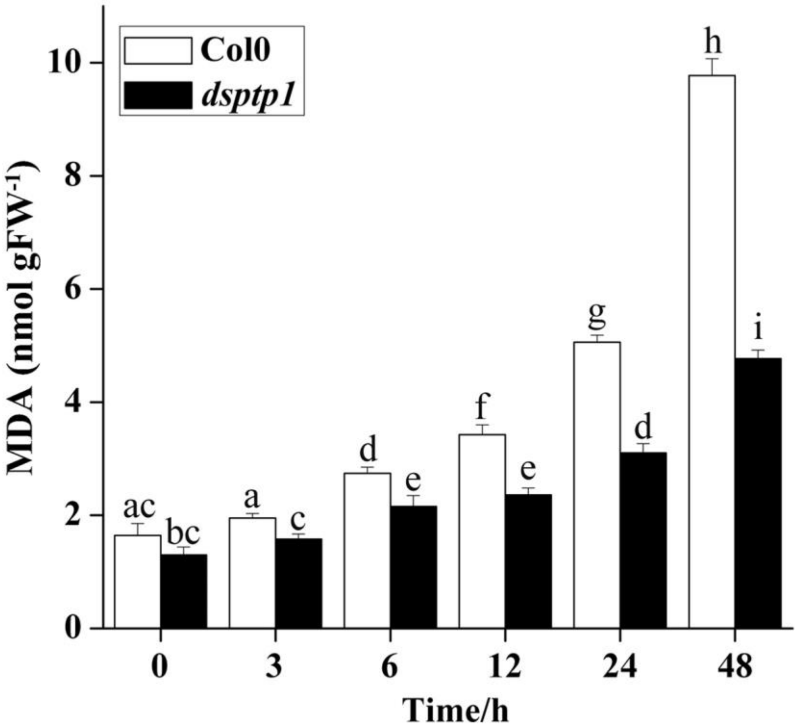

Supplementary Fig. S5. Comparsion of MDA content between WT and the *dsptp1* mutant under the treatment with 330 mM mannitol.

MDA content was determined in WT and *dsptp1* under 330 mM mannitol treatment. Means denoted by the same letter do not significantly differ at $P < 0.05$ according to Duncan's multiple range test. The experiments were repeated at least thrice with similar results.

Supplementary Table S1. Primers used for real- time RT-PCR assays.

Primer Name	Sequnence
AtActin2-FP	GTGAAGGCTGGATTTGCAGGA
AtActin2-RP	AACCTCCGATCCAGACACTGT
DsPTP1-FP	GTGTTCTTGTTCATTGCTTTGTTGG
DsPTP1-RP	GTCACTCACTTGCATAGACTTCTCG
NCED3-FP	GGTTTCTGGGAGATGGCTTGGT
NCED3-RP	ATGGCGGGAGAGTTTGATGATTG
CYP707A4-FP	GAAAGGAATACAGTACAGTC
CYP707A4-RP	GGATTAGATTTGGCTAACTAC
DREB2A-FP	GCAGACTATGGCTTAAATCAGGAC
DREB2A-RP	CTGAAACGGAGGTATTCCGTAGT
ICK1-FP	GATTGTTGTTGTAGCGGGAGG
ICK1-RP	TTCGTAACGTCCTTCTAATGGC
ABF1-FP	TTTCTTACTCCGTGCTGGCGT
ABF1-RP	GCTGTTTTGATTCGGCTGACC
COR15A-FP	TTCTTTCCACAGCGGAGCC
COR15A-RP	TGTTGCCGTCACCTTTAGCG
ERD1-FP	GCAGGCAAAAGCAGGATGAC
ERD1-RP	ATCAGGTCCCACCAGTATAGGC
ABI1-FP	GGTTCGATGTTAGATGGTCGG
ABI1-RP	GAGCATCGGTTTCTCCTTAGCT
ABI3-FP	ACATCTCCAGCTCCTGTCAACG
ABI3-RP	TGCACCAGAAGAGTCGTCACAG
ABI4-FP	AATCCGATTCCACCACCGAC
ABI4-RP	ACTTGCGAGTGCGCTTACGT
ABI5-FP	ATCAAGAACCGCGAGTCTGC
ABI5-RP	TCCAACTCCGCCAATGCA
